# Peripheral helper T cells, mavericks of peripheral immune responses

**DOI:** 10.1093/intimm/dxad041

**Published:** 2023-10-03

**Authors:** Hiroyuki Yoshitomi

**Affiliations:** Department of Immunology, Graduate School of Medicine, Kyoto University, Yoshida-Konoe-cho, Sakyo-ku, Kyoto 606-8501, Japan

**Keywords:** autoimmune, CXCL13, peripheral tissues, tertiary lymphoid structure, Tph cells

## Abstract

Peripheral helper T (Tph) cells have been established, through intensive efforts to elucidate local immune responses in human rheumatoid arthritis (RA), as a CD4 subset intimately involved in acquired immunity in peripheral tissues. Initially, Tph cells were noted as a CD4 population that produces high levels of CXCL13 in RA synovial tissues, followed by a demonstration of their ability to help B cells. In contrast to follicular helper T (Tfh) cells, Tph cells do not express the transcription factor BCL6 but express molecules such as CXCL13, interleukin (IL)-21, and inducible T-cell costimulator (ICOS) to help B cells in peripheral tissues. Subsequent studies showed that Tph cells are associated with various diseases, including autoimmune diseases, infections, and malignancies, and with the development of early life immunity. This review summarizes the phenotype and function of Tph cells in RA and discusses their differentiation and diversity in various conditions.

## Teaser

A comprehensive update of Tph cell phenotypes and functions

## Introduction

On the basis of involvement of autoantibodies and human leukocyte antigen (HLA) class II in autoimmune diseases (1), CD4^+^ T cells are thought to be at the forefront of the pathogenesis of autoimmunity, and attempts have been made to elucidate their role in autoimmune diseases. In the 2000s, the Th17 cell, a CD4 subset that produces the interleukin (IL)-17 family of cytokines, was shown to play a central role in various autoimmune mouse models, including autoimmune arthritis and autoimmune encephalomyelitis (2). Around this time, a paradigm shift in treating rheumatoid arthritis (RA) occurred with the advent of molecularly targeted drugs (3). The first molecularly targeted drug was infliximab, a chimera antibody targeting tumor necrosis factor (TNF), followed by the development of various drugs targeting molecules such as IL-17, IL-12/IL-23, IL-6R, CD80/CD86, and Janus kinases (JAKs), which are now used for the treatment of a variety of autoimmune diseases (3). Now, the therapeutic effects of these molecularly targeted drugs on disease have reaffirmed the role of the respective immune pathways in various human disorders (3).

The outcomes of these clinical studies suggested that although T cells are involved in human RA, the role of IL-17 and interferon-γ (IFN-γ) is not central in its pathogenesis. This raises the question of which CD4 subset is mainly involved in the etiology of human RA. Identification of peripheral helper T (Tph) cells by intensive attempts to elucidate local immune responses of RA as a CD4 subset answers this question. Tph cells can be defined as PD-1^hi^CXCR5^−^ CD4^+^ T cells that help B cells independent of BCL6 in peripheral tissues and that preferentially express certain signature genes of follicular helper T (Tfh) such as CXCL13, IL-21, and Inducible T-cell costimulator (ICOS). On the other hand, it is becoming clear that Tph cells exhibit diversity beyond this definition, depending on the disease and organ. This review summarizes the role of Tph cells in RA and then discusses the immunological functions, differentiation, and diversity of Tph cells under various conditions.

## Tph cells in RA

RA is an autoimmune disease characterized by autoantibodies such as rheumatoid factor and anti-citrullinated antibody (ACPA) and by systemic autoimmune articular inflammation accompanied by hyperplasia of synovial tissue, which leads to joint deformation and loss of the ability to live a full daily life (1). Several studies of RA synovial CD4^+^ T cells have contributed to the establishment of a distinct CD4 subset, the Tph cells. The first study related to Tph cells reported that a population of CD4^+^ T cells produces high levels of CXCL13 in synovial tissue, a region responsible for RA pathogenesis (4). Despite the prevailing notion that CXCL13 was mainly produced by Tfh cells, CXCL13-producing CD4^+^ T cells in RA synovium were negative for CXCR5 and BCL6 (4). A subsequent study showed that RA synovial CXCL13-producing CD4^+^ T cells are restricted to PD-1^hi^ and mostly CXCR5 negative (5). In addition, synovial CXCL13-producing CD4^+^ T cells in RA are almost independent of cells expressing IFN-γ, IL-17, or FoxP3, indicating that the synovial PD-1^hi^CXCR5^−^CXCL13^+^ CD4 population is a distinct CD4 subset (5). High expression of PD-1 (5) and CD69 (4, 5) implies that RA synovial CXCL13-producing cells are memory cells. Indeed, CXCL13-producing cells are in a memory state and rapidly secrete CXCL13 upon T cell receptor (TCR) re-stimulation (5). More importantly, RA synovial cells induce chemotaxis of B cells and CXCR5^+^ lymphocytes in a CXCL13-dependent manner (5), implying their involvement in the formation of tertiary lymphoid structures (TLSs).

Following these studies, synovial PD-1^hi^CXCR5^−^CD4^+^ T cells were found to provide help to B cells via IL-21 and the signaling lymphocytic activation molecule-5 (SLAM5) as effectively as Tfh cells do in RA (6). This B-cell help activity is preserved in circulating PD-1^hi^CXCR5^−^CD4^+^ T cells in the peripheral blood. PD-1^hi^CXCR5^−^CD4^+^ T cells were consistently increased in peripheral blood in autoantibody-positive (seropositive) RA but not in seronegative RA or psoriatic arthritis (6), strongly suggesting the involvement of Tph cells in RA pathogenesis. On the basis of these findings, PD-1^hi^CXCR5^−^CD4^+^ T cells were named Tph cells (6), providing help to B cells in the peripheral tissues. Tph cells express inflammation-related chemokine receptors such as CCR2, CCR5, and CX3CR1 instead of the chemokine receptor CXCR5 (6). Because of this chemokine receptor expression pattern, Tph cells function in peripheral tissues and Tfh cells function in lymph node germinal centers.

## Circulating Tph cells in diseases

Since many diseases, including autoimmune diseases, infections, and malignancies, involve inflammation in peripheral tissues, it is not surprising that Tph cells are involved in their pathogenesis. The association between various diseases and circulating Tph (cTph) cells in blood has been widely analyzed due to the accessibility of the peripheral blood. The definition of cTph cells is mainly PD-1^hi^CXCR5^−^ or PD-1^+^CXCR5^−^ memory CD4^+^ T cells. A similar approach has long been used with Tfh cells (7). Circulating Tfh (cTfh) cells have reduced BCL6 expression compared with lymph node Tfh cells but have preserved functions that provide help to B cells, and the frequency of cTfh cells is thought to be associated with systemic Tfh cell activity. cTph cells also have reduced CXCL13 and IL-21 expression compared with tissue Tph cells but also exhibit help to B cells (6, 8).

Diseases associated with increased peripheral cTph cells have been reported to include systemic lupus erythematosus (SLE) (9–12), Sjögren’s syndrome (13, 14), primary biliary cirrhosis (15), type I diabetes mellitus (16), IgG4-related diseases (IgG4-RD) (17, 18), systemic sclerosis (19), and IgA nephropathy (20). Furthermore, the association of cTph cell frequency with clinical indexes such as SLE disease activity index (SLEDAI) or anti-double-stranded DNA antibodies in SLE (9–11), worsening renal function in IgA nephropathy (20), insulin autoantibodies (IAA), islet tyrosine phosphatase 2 antibodies (IA-2A), and glutamic acid decarboxylase (GAD) autoantibodies in type I diabetes mellitus (16) has also been reported. Increases in cTph cells have also been reported in infectious diseases such as human immunodeficiency virus (HIV), chronic hepatitis B, and severe coronavirus disease 2019 (COVID-19) (21–23).

On the other hand, it is difficult to determine whether Tph cells are truly involved in the pathogenesis of the respective diseases from the analysis of peripheral blood alone. Because of shared differentiation mechanisms between Tph and Tfh cells (24), as will be discussed later, their frequencies in the peripheral blood are strongly correlated (25). Therefore, even in diseases where only Tfh cells are strongly involved in the pathology, the cTph cells and the disorders may appear related. Therefore, to elucidate the role of Tph cells in disease states, an investigation of local immune responses in the peripheral tissues is required.

## Tph cells and TLSs

Peripheral immune responses are closely related to the TLS, a discrete lymphoid structure similar to secondary lymphoid organs (SLOs) forming in peripheral tissues under chronic inflammation, autoimmune diseases, infections, and malignancies (26). Unlike SLOs, TLSs are not encapsulated. TLSs start as a lymphoid aggregate and continuously change into structures with primary follicles and eventually secondary follicles as they mature (27). In RA, lymphoid aggregates are found in most synovial tissues and mature TLSs with segregation of T cells and B cells (T–B segregation) in 20%–50% of cases (28). TLSs form lymphocyte niches in peripheral tissues, enhancing immune cell interaction and improving local antigen-presentation efficiency (27). Indeed, in RA, the disappearance of TLSs with molecularly targeted drugs is associated with a good prognosis (29); TLSs within malignancies are associated with a good patient prognosis in many types of cancer (30–32). Hence, the regulation of TLSs is crucial for immune responses in the peripheral tissues under various conditions.

CXCL13 is the most upregulated gene in CD4^+^ T cells of RA synovial tissues (33). Although there are reports that some human Th17 clones (34) or synovial Treg cells of juvenile idiopathic arthritis (35), in addition to Tfh cells, express CXCL13, single-cell RNA sequencing (scRNA-seq) of the entire RA synovial tissue shows that Tph cells are the main source of CXCL13 (36). Thus, although PD-1^hi^CXCR5^−^ is the most important phenotype in defining Tph cells, the preferential expression of CXCL13 is also an important feature of Tph cells. Despite the absence of direct data showing the induction of TLSs by Tph cells, the following findings collectively indicate that Tph cells play a crucial role in TLS induction: (i) ectopic CXCL13 is sufficient for TLS formation (37); (ii) scRNA-seq showing that Tph cells are the main source cells for CXCL13 (36); and (iii) RA synovial fluid mononuclear cells showed CXCL13-dependent lymphocyte migration *ex vivo* (5).

**Figure 1.**
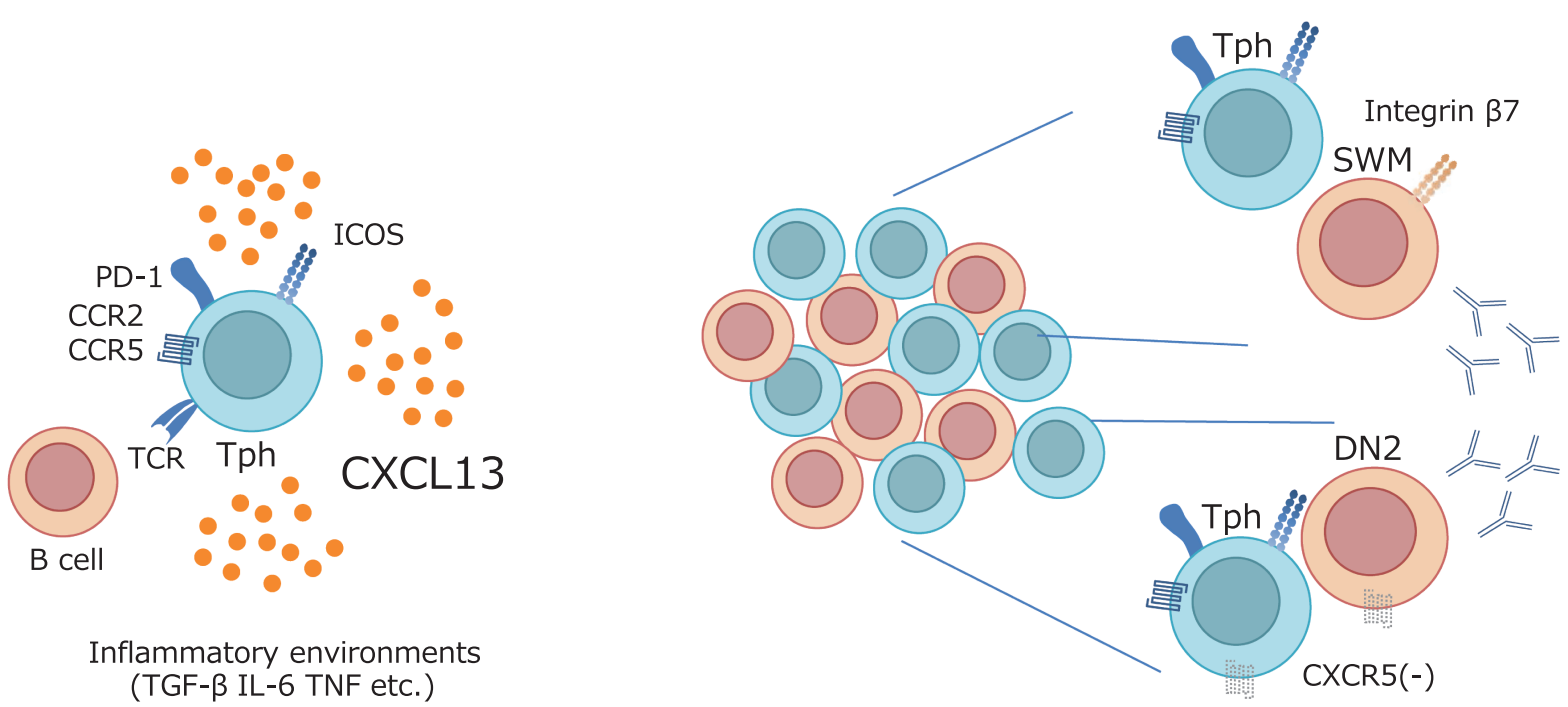
Tph cells vigorously secrete CXCL13 upon TCR stimulation and inflammation in peripheral tissues and trigger TLS formation (left). Within the TLS, especially in immature TLSs, Tph cells are intermingled with B cells in a mosaic-like fashion and provide help to neighboring B cells. The coexistence of Tph and B cells is regulated by chemokine receptor expression patterns and integrins (right).

**Figure 2.**
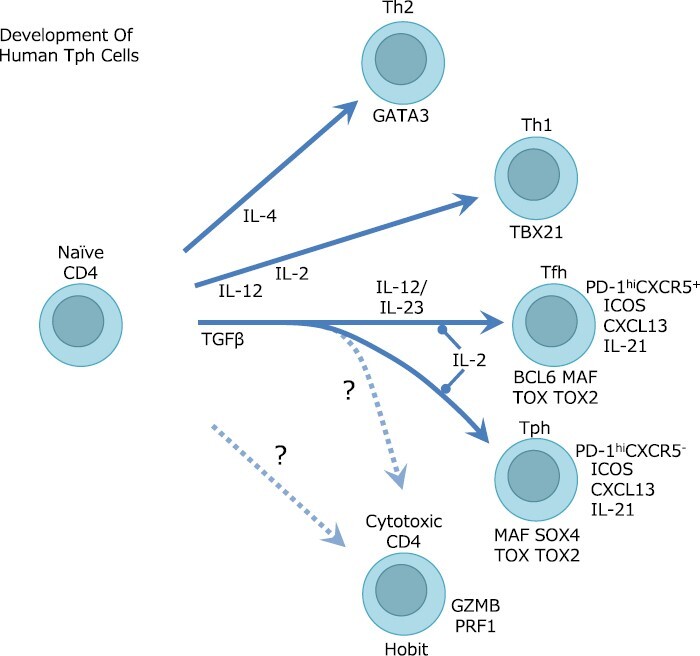
Classically, human naive CD4^+^ T cells differentiate into Th1 cells by IL-12 and Th2 cells by IL-4, and the key transcription factors are TBX21 and GATA3, respectively. TGF-β plays an essential role in the differentiation of both Tph and Tfh cells. The differentiation of human Tfh cells requires IL-12/IL-23-mediated STAT3/STAT4 stimulation, which is involved in the expression of the master transcription factor BCL6, IL-21, PD-1, and CXCR5. Tph cells also express PD-1, CXCL13, IL-21, and ICOS, which are Tfh cell’ features but do not express CXCR5 or BCL6. SOX4 contributes CXCL13 expression under TGF-β conditions. MAF plays an essential role in the B cell help activity of Tph cells as well as Tfh cells. TOX and TOX2 are involved in the expression of checkpoint molecules such as PD-1 expressed by Tfh and Tph cells. Cytotoxic CD4 cells express the transcription factor Hobit and cytotoxic molecules such as GZMB and PRF1. The differentiation pathway of cytotoxic cells CD4 is unclear, but studies on RA synovial CD4 cells suggest a possible link with Tph cell differentiation.

TCR stimulation is an important trigger to produce CXCL13, and it is known that inflammatory cytokines and TGF-β in the environment are involved in persistent CXCL13 secretion (5, 38, 39) (Fig. 1, left). On the other hand, activation of the IL-2/STAT5 pathway and downstream Blimp1 expression suppress CXCL13 (38, 39) (Fig. 2). Presumably, this suppression would be a negative regulation that attempts to stop TLS formation once sufficient acquired immunity has begun to function. Reports of TLS reduction following TNF-inhibitor therapy (29) fit with the involvement of inflammatory cytokine production in persistent CXCL13 by Tph cells (5). In diseases other than RA, CXCL13 has been reported to be produced by Tph cells in the intestine of ulcerative colitis (UC) (40) and in malignant tumors (41–43). In malignancies, CD8 T cells also produce CXCL13 (44, 45). Interestingly, CXCL13^+^CD4^+^ T cells but not CXCL13^+^ CD8 T cells accumulate in early TLSs of ovarian cancers whereas, in the matured TLSs with T–B segregation, CD21^+^ follicular dendritic cell (FDC)-like stroma cells, rather than CD4^+^ T cells, expressed CXCL13 (44).

These findings imply that Tph cells play an important role in the early stages of TLS formation and, as the TLS matures, FDCs arise within the TLS to form discrete zones. Thus, Tph cells induce TLSs in organs where lymphoid tissue is not originally present, creating a lymphocyte niche where antigens can be rapidly presented, and immune cells can intensively interact locally.

## B-cell helper activity of Tph cells

One of the important functions of Tph cells as CD4^+^ helper T cells is providing help to B cells. As Tph cells do not express CXCR5, they do not migrate to the germinal centers of SLOs, such as lymph nodes and tonsils, but they can coexist in a mosaic-like fashion with B cells in the immature TLSs, which are dominant in inflammatory tissues. Eventually, Tph cells provide help to the coexisting B cells (5, 6, 40) (Fig. 1, right). In mature TLSs with T–B segregation, CXCR5-expressing Tfh cells may infiltrate the B-cell zone and exert their function.

Tph cells, via IL-21 and SLAM family molecules, help memory B cells as efficiently as Tfh cells do (6). On the other hand, Tph cells do not appear to exert sufficient B-cell helping activity for naive B cells. When Tph or Tfh cells were co-cultured with naive B cells, Tfh cells induced a class-switch to IgG or IgA in B cells, but co-culture with Tph cells did not induce a class-switch (8). This implies that Tfh and Tph cells differ in their localization and the nature of their B-cell helper activity. The difference in immunological significance may be that Tfh cells are responsible for inducing new high-affinity antibodies with frequent somatic hypermutation (SHM), whereas Tph cells rapidly help already activated memory B cells.

These differences may be due not only to differences in the properties of Tfh and Tph cells but also to differences in the lymphatic structures in which they reside. In the germinal centers of SLOs, organized zones such as the light zone and the dark zone contribute to high-affinity antibody differentiation with high SHM (46). On the contrary, T and B cells in inflammatory tissues are frequently intermingled in a mosaic-like fashion (5, 6, 40), and B cells would develop less specifically. As might be expected from this, there is less SHM in Tph cell-associated B-cell differentiation.

In UC, for example, Tph cells and switched memory B (SWMB) cells are increased and found adjacent in the intestinal intestine (40). Interestingly, a comparison of SHM in SWMB cells in normal and UC intestines showed a significant reduction in SHM in the SWMB cells from patients with UC (40). Furthermore, a decrease in SHM is also observed in peripheral blood SWMB cells expressing integrin β7, an integrin important for homing to the gut, compared with healthy individuals (40) (Fig. 1, right).

In RA, the SHM of peripheral blood IgG is also lower than in healthy controls (47). This also suggests that B-cell differentiation with low SHM occurs in RA, where Tph cells dominate. Interestingly, ACPA, a specific autoantibody for RA that arises before RA onset (48), has a high SHM (49). This suggests that before RA onset, when Tph cells are inactive, Tfh cells dominantly support the establishment of ACPA.

In SLE, CD11c^+^ B cells are candidate target B cells for Tph cells; CD11c^+^ B cells, also known as double negative 2 (DN2) B cells or age-associated B cells (ABCs), do not express IgD, CD27, or CXCR5 and are supposed to differentiate extrafollicularly (50) (Fig. 1, right). Tph cells and DN2 B cells, both of which do not express CXCR5, localize extrafollicularly and may coexist. Although no reports directly showed the coexistence of Tph cells and DN2 B cells in organs, cTph cell numbers correlate well with CD11c^+^ B cells in SLE peripheral blood (11). Consistently, DN2 B cells have less SHM than other fractions do (50). In severe COVID-19, Tph cells and DN2 B cells also increase in the peripheral blood, and SHM is low in DN2 B cells (23, 51). Upregulation of autoantibodies such as anti-nuclear antibody and ACPA in COVID-19 patients supports the physiological significance of Tph and DN2 cells in COVID-19 patients (52).

Thus, Tph cells may exhibit a B-cell-helping activity different from Tfh cells and may be associated with rapid but less specific B-cell differentiation rather than antibody development with high affinity and high SHM. In addition, the B cells targeted by Tph cells vary according to diseases and local environment. The coexistence of the B cells and Tph cells is regulated by the expression pattern of molecules such as integrins and chemokine receptors, which control the migration and localization of cells, and the key factors restraining the coexistence vary according to disease and organs. The immunological significance of such B-cell helper activity of Tph cells remains to be determined.

## Tph cell differentiation

Depending on the cytokine environment, CD4^+^ T cells differentiate into multiple types of effector cells (Fig. 2). The dominance of Tph cells in inflammatory peripheral tissues also implies that the cytokine milieu is associated with Tph cell differentiation. Indeed, *in vitro* T-cell differentiation systems provide insights into the differentiation mechanisms of Tph cells. Interestingly, studies of Tph and Tfh differentiation have been performed independently at about the same time and have revealed many similarities between human Tph and Tfh cell differentiation. The most important signal for human Tph and Tfh cell differentiation is TGF-β signaling (7, 38, 53) (Fig. 2). It is reported that TGF-β1, TGF-β2, and TGF-β3 induce Tph cells *in vitro* (38), while it is reported that TGF-β1 (7) and activin A (53) induce Tfh cells. Adding TGF-β signaling along with TCR stimulation induces CXCL13 and PD-1 expression in human CD4^+^ T cells. *In vitro* stimulation of human CD4^+^ T cells with TGF-β induces the expression of transcription factor SOX4, leading to intensive CXCL13 expression by PD-1^hi^CXCR5^−^ cells (39). On the other hand, the transcription factor MAF induces the expression of molecules such as ICOS, IL-21, and the SLAM family in Tph cells and plays crucial roles in B-helper activity (11, 39). In Tfh differentiation, activating STAT3 and STAT4 by IL-12/IL-23 stimulation induces expression of Tfh-associated molecules, including BCL6, IL-21, PD-1, and CXCR5, in concert with TGF-β-induced SMAD2/SMAD3 (7, 53). Although what separates the fate of Tph and Tfh cells is still unclear, IL-12/IL-23 signaling at early stages of differentiation may be important for Tfh differentiation. Consistently, therapies targeting IL-12/IL-23 are ineffective in RA, suggesting that IL-12/IL-23 does not play a central role in Tph cell differentiation or function (3, 54). Considering that IL-12 and IL-2 play a positive role in Th1 differentiation and TGF-β plays a negative role (55), it also can be said that Th1 and Tph cell differentiation are regulated by opposing microenvironments (Fig. 2).

A partially shared TCR between Tph cells and Tfh cells in lymph nodes of HIV patients supports the common differentiation process of these cells (21). Tfh and Tph cells share the expression of the checkpoint molecules PD-1 and TIGIT and the expression of transcription factors TOX and TOX2 (6, 56–58) (Fig. 2), which are known to be expressed by exhausted cytotoxic CD8 T lymphocytes (CTLs) and may be involved in the expression of checkpoint molecules such as PD-1, CTLA-4, and TIGIT as well as CTLs (39, 59). On the other hand, unlike exhausted CTLs, Tph, and Tfh cells can perform immune functions despite negative signals provided by PD-1 (60). Thus, antigenic stimuli and the local inflammatory environment play an important role in Tph cell differentiation, implying the diversity of Tph cells depending on conditions and organs.

## Diversity of Tph cells

The exhaustion marker PD-1 is strongly expressed on cells that have experienced strong antigenic stimulation (61). Therefore, various cell types that have experienced antigenic stimulation can be categorized as PD-1^hi^CXCR5^−^. As mentioned above, the importance of the microenvironment for Tph cells also suggests disease-specific and organ-specific diversity of Tph cells. Indeed, advances in scRNA-seq analysis reveal differences in Tph cell function in different conditions and organs. A comparison of skin and kidney Tph cells from SLE patients also showed elevated activation signatures, including the expression of HLA molecules, ICOS, and cytokines in the kidney Tph cells (62).

Furthermore, a comparison of cutaneous lymphocyte signatures between systemic sclerosis and SLE patients showed increased interferon-induced signatures such as *MX1, IFI44L*, and *IFIT3*, and activation and exhaustion-associated genes such as *PDCD1, TOX, LAG3, TNFRSF18*, and *MAF* in SLE Tph cells (62). Some reports suggest that type I/III interferons may be involved in Tph cell differentiation (11, 12), and the relevant signaling pathways and transcription factors are expected to be elucidated. In addition to the Tph cells that provide B-cell help via IL-21/MAF, another PD-1^+^CXCR5^−^ population also provides B-cell help via IL-10 and succinate in SLE (63).

Interestingly, unsupervised clustering in RA synovial scRNA-seq places Tph and Tfh cells in the same cluster (33). Although technical limitations of RNA detection by scRNA-seq may have prevented the detection of their differences, this implies that Tph and Tfh cells have fairly similar gene expression profiles in the same microenvironment. It has also been reported that Tph cells produce cytotoxic molecules such as perforin, granzymes, and GPR56 (64). A similar cytotoxic phenotype was observed in Tph cells of IgG4-RD (18). The role of these cytotoxic molecules in autoimmune pathology still needs to be determined.

Tph cells appear to be generated even in healthy young individuals. Bronchus-associated lymphoid tissue (BALT) exists around the lung airway of infants and young child under 3 years old, but its number decreases after that (65). PD-1^hi^CXCR5^−^CD4^+^ cells exist here and are considered to be involved in B cell help in the lung to establish infant-acquired immunity (65). These findings suggest CD4^+^ T cells with a Tph feature develop even under physiological conditions. This differentiation program disappears once after 3 years but may sometimes start to work in joints, tumors, and infections, depending on tissue environments. The B cells from lung TLSs of young individuals are no less active than those of the lymph nodes, undergoing SHM and class-switching. Interestingly, lung naive B cells express less CXCR5 than those of lymph nodes, implying that lung Tph cells and CXCR5^−^ B cells preferentially interact. Although lung PD-1^+^CXCR5^−^ CD4^+^ T cells express BCL6 at a low level, BCL6 is upregulated in the ICOS^+^TIGIT^+^ fraction of lung Tph cells to a level equivalent to lymph node Tfh cells (65). The similarities and differences between lung Tph cells of young children and Tph cells in disease need further clarification.

T cells that exhibit Tph cell features also play essential roles in tumors. First, certain types of lymphomas have Tph cell signatures. Classic Hodgkin lymphoma (CHL) shows a phenotype of CD4^+^PD-1^+^CXCL13^+^CXCR5^−^ in the lymphocyte-rich classic type (66) and also expresses TOX and TOX2 (67). On the other hand, most angioimmunoblastic T-cell lymphoma (AITL) cases express Tfh signature genes such as BCL6 in addition to PD-1 and CXCL13. These findings suggest that Tph and Tfh signatures can each be distinct stable epigenomic states.

In addition, CD4^+^ T cells with a Tph signature play crucial roles in tumor immunity (68). Although Tfh cells have been reported to produce CXCL13 in various cancer types, in breast cancer, so-called TfhX13 cells (Tfh-like cells that express CXCL13 and PD-1 but are negative for CXCR5) are associated with TLS formation (42, 43). Using a definition of PD-1^hi^CXCR5^−^, TfhX13 cells are considered to be equivalent to Tph cells. In hepatocellular cancer, it has been reported that PD-1^+^CXCL13^+^CD4^+^ T cells form niches and prime CTLs (69). Expansion of HCC Tph cells by immune checkpoint inhibitors suggests that Tph cells can expand locally upon exposure to local antigens (69). TGF-β in the tumor microenvironment (70) is also involved in the differentiation of cells similar to Tph cells in autoimmune diseases within cancer tissue. Interestingly, the production of CXCL13 by Tfh and Tph cells varies by tumor type and organ. Furthermore, there are some tumor types in which CXCL13 expression is associated with a favorable prognosis for the patient and others in which CXCL13 expression is associated with a poor prognosis. Thus, even in malignancies, Tfh and Tph cells show diversity depending on tumor type and organ (68).

## Tph cells between humans and mice

Good mouse models of human Tph cells are essential for further understanding the function and ontogeny of Tph cells. In mice, it has been reported that PD-1^+^CXCR5^−^^/dim^ cells appear in the lymphopenia-induced proliferation (LIP) system after transplanting CD25^−^ CD4^+^ T cells into nude mice (71). These cells help B cells in an IL-21 and ICOS-dependent manner, similar to human Tph cells, but unlike human Tph cells, they strongly express BCL6. Interestingly, in this system, PD-1^+^ cells migrate to the germinal center even though they do not express CXCR5. Considering that human Tph cells do not express BCL6 at a high level and are located in the perifollicular area, this might not be a good model of human Tph cells. There have been reports of PD-1^hi^CXCR5^−^ Tph-like cells associated with TLSs in aging mouse kidneys, and the absence of TLS formation when CD30L is deleted, suggesting that CD30L plays an important role in TLS formation by murine T cells (72). The upregulation of *Cxcl13* mRNA in mouse PD-1^+^CD4^+^ T cells is preserved (72), but there does not appear to be a vigorous production of CXCL13 protein by mouse T cells (73). Satb1 is associated with such limited production of CXCL13 in mice. Satb1-deficient Tfh cells produce CXCL13 intensively in mouse tumors (74). Thus, a good mouse model of human Tph does not yet exist, and further improvement of mouse models is desired.

## Ontogeny of Tph cells

The ontogeny of Tph cells is an intriguing issue both pathologically and immunologically. Tph cells are a fraction identified from human samples, and there is so far no good mouse model for human Tph cells yet. Therefore, it is reasonable to elucidate their ontogeny in humans, but various constraints of human samples limit the data related to ontogeny. A possible clue related to the ontogeny of Tph cells is that Tfh cells and Tph cells partly share TCR sequences strongly in the lymph nodes of HIV patients (21). Because of the huge diversity of TCR sequences, they can be treated as cell barcodes. Sharing a TCR between Tfh cells and Tph cells suggests that these cells differentiate from a common cell. Therefore, in the case of HIV patients, it is reasonable to speculate that naive CD4^+^ T cells start differentiation by receiving antigen presentation from DCs in lymph nodes, and Tfh cells and Tph cells differentiate after chronic antigen stimulation.

Since, Tph cells in the infant lungs share clones with lymph nodes just partially (65), it is reasonable to speculate that these cells differentiate along with the maturation of BALT from the resident cells of the lung. Some clones that overlap with lymph nodes may differentiate across both the lung and the lung lymph nodes.

In diseases such as RA and tumors, T cell differentiation can occur in peripheral tissues with TLS where antigens can be presented. The origin of Tph cells in these tissues, whether derived from circulating or resident cells, may depend on the organ. For example, normal synovium is a thin membrane-like tissue composed of two to three cell layers and has few lymphocytes in the sublining (75), implying that most synovial Tph cells likely originate from circulating cells. On the other hand, in cancers in lymphocyte-rich tissues such as lungs, tissue-resident cells are more likely to be the origin of Tph cells.

Similar to HIV studies, TCR analysis of tissue T cells has been performed. The precursor-like Tph cells of RA synovial fluid were reported to share TCRs strongly with activated Tph cells (64). This finding implies that the differentiation of Tph cells from precursor to activated state occurs within the tissue. Furthermore, Tph cells and the CD4 fraction expressing cytotoxic molecules with a transcription factor Hobit also share to some extent, implying that Tph cells and cytotoxic CD4 may arise from a common precursor cell within the synovium (64) (Fig. 2). Of course, if all these fractions circulate frequently, we can see TCR sharing among fractions in the synovium, even if they differentiate in lymph nodes or elsewhere. If there is a large difference in the clones that proliferate oligoclonally between different joints (left and right, etc.) of the patient, it would suggest independent differentiation within the joint after a certain step. High-resolution TCR analysis of physically distant sites in the same patient with autoimmune or malignant diseases is expected to provide some answers about ontogeny.

## Conclusions

Fifteen years after the first report related to Tph cells and 5 years after the naming of Tph cells, it has become increasingly clear that Tph cells are involved in various diseases. Furthermore, advances in scRNA-seq have revealed their diversity and intensive investigation of human samples revealed their existence in healthy infant lungs. On the other hand, their molecular mechanisms, roles in disease and early life, and their ontogeny remain to be clarified. A further understanding of the phenotype of their variety and their regulation by signaling pathways and transcription factors is expected to lead to a more appropriate classification of Tph cells and a better understanding of their roles in the pathophysiology of various diseases.
